# Usefulness of suPAR as a biological marker in patients with systemic inflammation or infection: a systematic review

**DOI:** 10.1007/s00134-012-2613-1

**Published:** 2012-06-16

**Authors:** Yara Backes, Koenraad F. van der Sluijs, David P. Mackie, Frank Tacke, Alexander Koch, Jyrki J. Tenhunen, Marcus J. Schultz

**Affiliations:** 1 Department of Intensive Care Medicine, Academic Medical Center, University of Amsterdam, Meibergdreef 9, 1105 AZ Amsterdam, The Netherlands; 2 Laboratory of Experimental Intensive Care and Anesthesiology, Academic Medical Center, Meibergdreef 9, 1105 AZ Amsterdam, The Netherlands; 3 Department of Anesthesiology, Red Cross Hospital, Vondellaan 13, 1942 LE Beverwijk, The Netherlands; 4 Department of Medicine III, RWTH-University Hospital, Pauwelsstrasse 30, 52074 Aachen, Germany; 5 Critical Care Medicine Research Group in Department of Intensive Care Medicine, Tampere University Hospital, PO BOX 2000, 33521 Tampere, Finland; 6 Department of Surgical Sciences/Anaesthesiology and Intensive Care Medicine, Uppsala University, PO BOX 256, 751 05 Uppsala, Sweden

**Keywords:** Soluble uPAR, Soluble urokinase plasminogen activator receptor (suPAR), Biomarker, Intensive care, Critical illness, Sepsis

## Abstract

**Purpose:**

Systemic levels of soluble urokinase-type plasminogen activator receptor (suPAR) positively correlate with the activation level of the immune system. We reviewed the usefulness of systemic levels of suPAR in the care of critically ill patients with sepsis, SIRS, and bacteremia, focusing on its diagnostic and prognostic value.

**Methods:**

A PubMed search on suPAR was conducted, including manual cross-referencing. The list of papers was narrowed to original studies of critically ill patients. Ten papers on original studies of critically ill patients were identified that report on suPAR in sepsis, SIRS, or bacteremia.

**Results:**

Systematic levels of suPAR have little diagnostic value in critically ill patients with sepsis, SIRS, or bacteremia. Systemic levels of suPAR, however, have superior prognostic power over other commonly used biological markers in these patients. Mortality prediction by other biological markers or severity-of-disease classification system scores improves when combining them with suPAR. Systemic levels of suPAR correlate positively with markers of organ dysfunction and severity-of-disease classification system scores. Finally, systemic levels of suPAR remain elevated for prolonged periods after admission and only tend to decline after several weeks. Notably, the type of assay used to measure suPAR as well as the age of the patients and underlying disease affect systemic levels of suPAR.

**Conclusions:**

The diagnostic value of suPAR is low in patients with sepsis. Systemic levels of suPAR have prognostic value, and may add to prognostication of patients with sepsis or SIRS complementing severity-of-disease classification systems and other biological markers.

## Introduction

Systemic levels of soluble urokinase plasminogen activator receptor (suPAR), a protein derived from cleavage and release from the cell membrane-bound urokinase plasminogen activator receptor (uPAR), positively correlate with the activation level of the immune system. Numerous observational studies have shown systemic levels of suPAR to be increased with cancer [1, 2], and various infectious and inflammatory diseases, including infections with human immunodeficiency virus (HIV) [3], malaria [4, 5], tuberculosis [6, 7], central nervous system infections [8], arthritis [9, 10], liver fibrosis [11], and inflammatory bowel disease [12]. In addition, systemic levels of suPAR have been shown to have prognostic value in predicting the severity and outcome in patients with cancer [13, 14]. Systemic levels of suPAR were also found to have a strong prognostic value in HIV-infected individuals [15, 16].

Systemic levels of suPAR are also increased in critically ill patients [17]. However, the usefulness of suPAR as a biological marker in critical illness is uncertain. The aim of this systematic review is to provide an overview of studies investigating the diagnostic and prognostic properties of suPAR in critically ill patients with sepsis, systemic inflammatory response syndrome, or bacteremia. We hypothesized that suPAR has both diagnostic and prognostic value in these inflammatory conditions. We also hypothesized that systemic concentrations of suPAR correlate with severity-of-disease classification system scores and other biological markers of severity of disease. Finally, we were interested in changes in systemic levels of suPAR after initiation of treatment to see whether suPAR has any potential for use in guiding therapy.

## Materials and methods

### Data sources

Two methods were used to identify relevant papers on suPAR as a biological marker in critically ill patients in the medical literature. First, an electronic search in the PubMed database was performed. Searches were also performed using the Cochrane Library and the Cochrane Database of Systematic reviews. Second, reference lists of identified and selected papers were reviewed for studies not identified with our search. Searches were restricted to original studies in humans and manuscripts written in English.

### Keywords (text word)

The following keywords were used, alone or in combination, to identity relevant papers: (1) condition (“critical care” or “intensive care”), (2) subject (“human”), (3) test (“suPAR” or “soluble urokinase plasminogen activator receptor” or “soluble uPAR” or “soluble uPA receptor” or “soluble urokinase PA receptor” or “cleaved urokinase plasminogen activator receptor” or “cleaved uPAR” or “cleaved uPA receptor” or “cleaved urokinase PA receptor” or “cleaved CD87” or “soluble CD87”), and (4) disease (“systemic inflammation,” “SIRS,” “bacteremia,” “sepsis,” or “septic shock”).

### Study selection

Titles and abstracts were reviewed, and papers that reported on studies of suPAR in critically ill patients with systemic inflammation or infection were selected. Thus, only papers on studies of critically ill patients were included (i.e., studies of patients who were admitted to an intensive care unit or to a hospital with systemic inflammation or infection). In case of uncertainty the complete paper was obtained and evaluated. Inclusion of papers was not restricted by methodological quality or any other critically appraisal criteria other than the criteria formulated for data extraction.

### Quality assessment

The quality of the included studies was evaluated by applying the 25-item criteria developed by the Standards for Reporting of Diagnostic Accuracy (STARD) committee [18, 19]. The maximum quality score that could be given to a study was 25 points over five categories. For each category, results were derived from consensus among three reviewers (Y.B, K.S and A.K).

### Data extraction

Manuscripts were criticized along the following four subjects: (1) is suPAR a useful diagnostic marker in detection of infection, (2) is suPAR a useful prognostic marker in patients with SIRS, bacteremia or sepsis, (3) do systemic concentrations of suPAR correlate with disease severity scores and markers of organ failure in these patients, and (4) how do systemic levels of suPAR respond to initiation of treatment?

## Results

### Search results

The search performed in May 2011 in the PubMed database revealed nine papers as original studies [17, 20–27]. One additional paper was found in the reference list of identified and selected papers [28]. The Cochrane Library and the Cochrane Database of Systematic reviews revealed no reviews or meta-analyses of suPAR in critically ill patients. Quality evaluation of the included studies using the STARD checklist is presented in Table 1. For 181 of the in total 250 items, complete agreement was observed between reviewers.

**Table 1:** Quality evaluation of the included studies using the Standard for Reporting of Diagnostic Accuracy (STARD) checklist [18, 19]

Study	References	Years	Title/abstract/keywords	Introduction	Methods	Results	Discussion	Total
Maximum score for each category^a^			1	1	11	11	1	25
Mizukami	[17]	1995	1	0	6	3	0	10
Molkanen	[20]	2011	1	1	8	8	1	19
Moller	[21]	2006	1	1	7	6	1	16
Kofoed	[22]	2007	1	1	10	8	1	21
Kofoed	[23]	2008	1	1	8	7	1	18
Kofoed	[24]	2006	0	1	7	3	1	12
Koch	[25]	2011	1	1	8	6	1	17
Wittenhagen	[26]	2004	1	1	7	5	1	15
Huttunen	[27]	2011	1	1	8	8	1	19
Florquin	[28]	2001	0	1	6	4	0	11

^a^For each category, results are derived from consensus among three reviewers as the number of items from the checklist present in the original article

Study populations in the retrieved studies comprised patients with sepsis and/or septic shock, SIRS and bacteremia. Importantly, definitions for diagnoses varied among the studies. Table 2 presents the criteria used for diagnosis of each study. Of note, two studies by Kofoed et al. [22, 23] used the same cohort and data set to investigate either the diagnostic [22] or the prognostic [23] value of suPAR.

**Table 2:** Patients characteristics of the included studies

Study	References	Criteria used for diagnosis	Diagnosis on admission as described by the authors (no. of patients)	Type of test used
Mizukami	[17]	A recognized source of infection or a hemodynamic profile suggestive of sepsis, along with fever, granulocytosis, and/or respiratory failure requiring ventilatory support	Clinical sepsis syndrome (13)	ELISA
Molkanen	[20]	Blood culture positive for *S. aureus*	Bacteremia (59)	ELISA
Moller	[21]	Blood culture positive for *S. pneumoniae*	Bacteremia (128)^a^	ELISA
Kofoed	[22]	At least two SIRS criteria^b^	SIRS (151)	Luminex multiplex assay
Kofoed	[23]	At least two SIRS criteria^b^	SIRS (151)	Luminex multiplex assay and ELISA
Kofoed	[24]	Blood culture positive for *Pneumococcus pneumonia* or *E. coli* and at least two SIRS criteria	Bacterial sepsis (10)	Luminex multiplex assay and ELISA
Koch	[25]	Severe sepsis and septic shock criteria^b^	Severe sepsis and septic shock (197)	ELISA
Wittenhagen	[26]	Blood culture positive for *S. pneumoniae*	Bacteremia (141)	ELISA
Huttunen	[27]	Blood culture positive for *S. aureus*, *S. pneumonia*, β-haemolytic *streptococcus* or *E. coli*	Bacteremia (132)	ELISA
Florquin	[28]	Acute symptoms of urinary tract infection, pyuria, urine gram staining with gram-bacteria, and metabolic or hematologic signs of systemic infection, including two of the three indicators: tachycardia, leukocytosis or fever	Urosepsis	ELISA

^a^A total of 133 patients were included in this study. Data for suPAR were accessible from 128 of the patients

^b^Criteria as recommended and defined in the American College of Chest Physicians/Society of Critical Care Medicine (ACCP/SCCM) Consensus Conference [29]

Seven studies evaluated the diagnostic value of suPAR [17, 22, 24–28]; six studies investigated the prognostic value of suPAR [20, 21, 23, 25–27]. Three studies correlated systemic levels of suPAR to disease severity scores [25, 27, 28]; three studies investigated changes of systemic levels of suPAR after initiation of treatment [25, 27, 28].

In addition, the authors of two publications [25, 26] kindly provided their raw data, which were used for re-drawing figures.

### Diagnostic value of suPAR

**Fig. 1:**
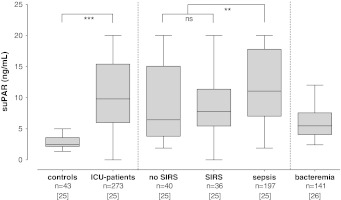
Systemic levels of suPAR in healthy controls and critically ill patients with SIRS or sepsis, and patients with bacteremia. Systemic levels of suPAR are significantly higher in patients with sepsis, as compared to patient without sepsis or patients with SIRS. Data represent medians with their interquartile range. Extremes were excluded from the figure. *Stars* indicate the level of statistical difference. Reproduced with permission from [25] and [26]

**Fig. 2:**
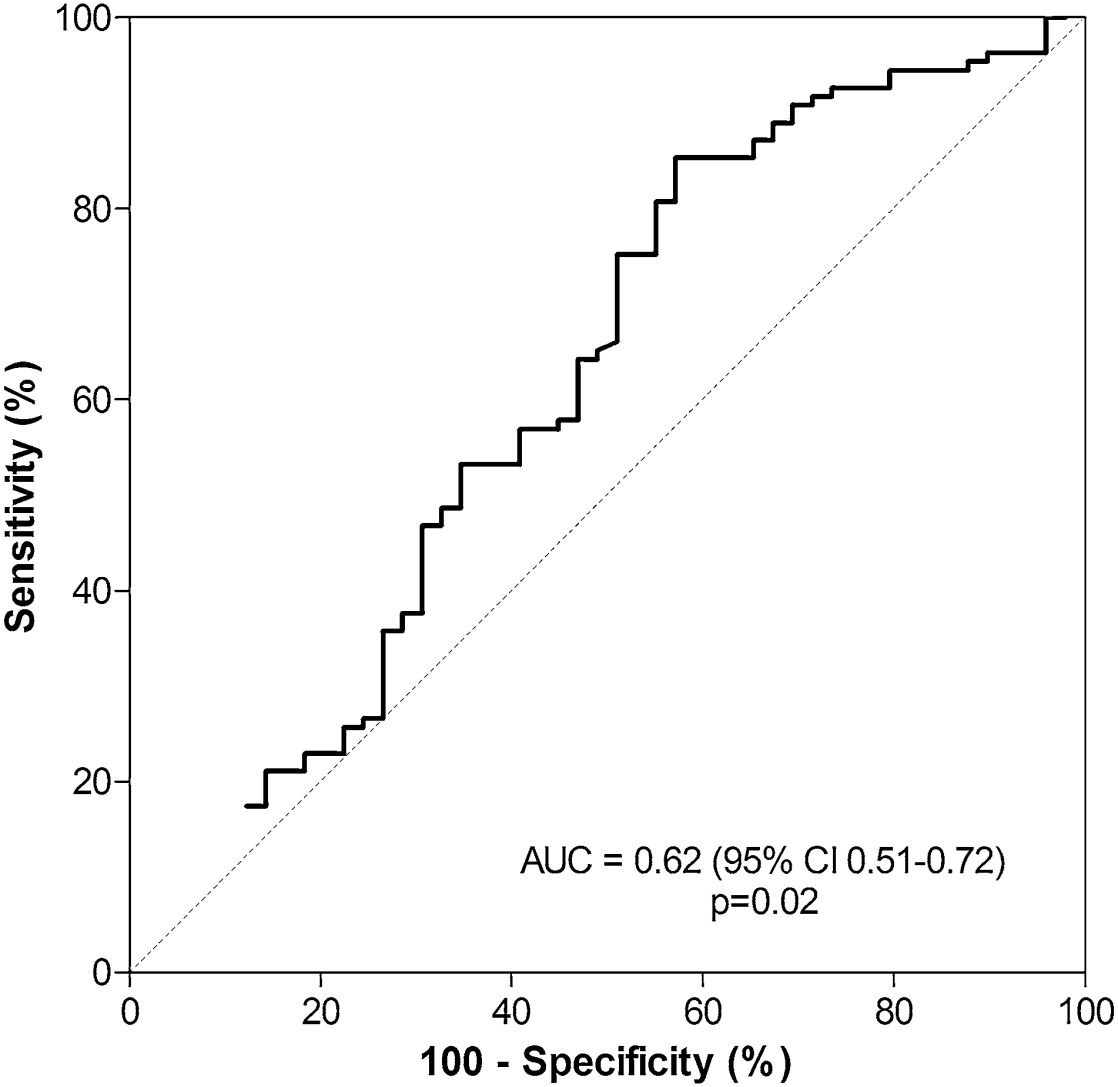
Diagnostic power of suPAR. ROC curve analysis showing the diagnostic power of systemic levels of suPAR in predicting sepsis on admission. AUC, area under the curve. The *p* value indicates the level of statistical significance. Adapted with permission from [25]

Systemic levels of suPAR were significantly higher in critically ill patients, compared to healthy controls [17, 24–26, 28]. A gradual increase in levels of suPAR was seen from critically ill patients who did not fulfill SIRS criteria to patients with SIRS and patients with sepsis (Fig. 1) [25]. However, the area under the receiver-operating characteristic curve (AUC) for suPAR to discriminate between non-septic and septic ICU patients is reported to be poor (Fig. 2) [25].

Systemic levels of suPAR were significantly higher in patients with blood culture-positive bacteremia compared to healthy controls [26]. Levels of suPAR in patients with gram-positive bacteremia did not appear to be different from those with gram-negative bacteremia [27]. Also, suPAR seemed to have no value in discriminating patients with bacterial infection from patients with viral or parasitic infections [22]. Finally, levels of suPAR have been shown to be similar in patients with pulmonary sepsis and in those with extra-pulmonary sepsis [25].

Compared to other frequently used biological markers that can be measured easily with commercially available kits, including C-reactive protein (CRP), procalcitonin (PCT), and soluble triggering receptor expressed on myeloid cells-1 (sTREM-1), suPAR added little to the diagnostic process [22, 25].

### Prognostic value of suPAR

**Fig. 3:**
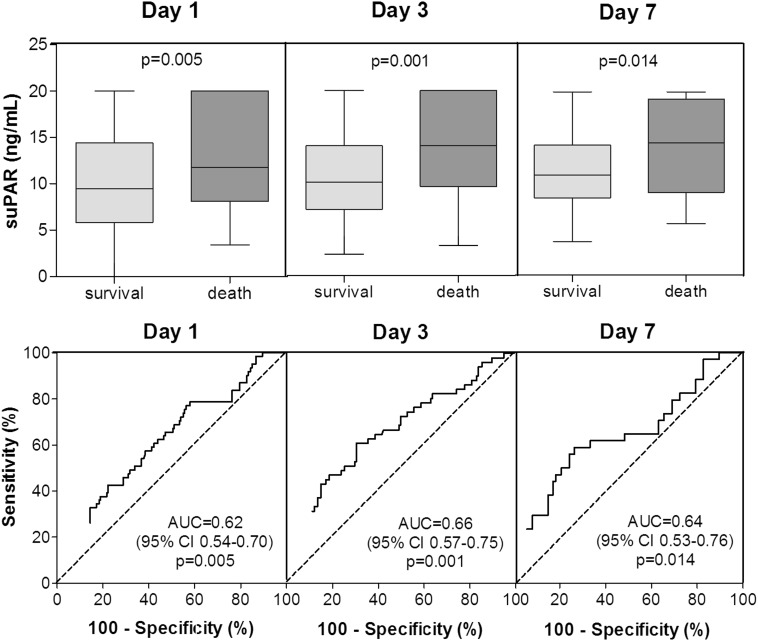
Prognostic power of suPAR in ICU patients. Box plot graphics and ROC curve analyses showing the prognostic power of suPAR for mortality on admission, and day 3 and 7 after admission in ICU patients. AUC, area under the curve. The P–value indicates the level of statistical significance. Adapted with permission from [25]

**Fig. 4:**
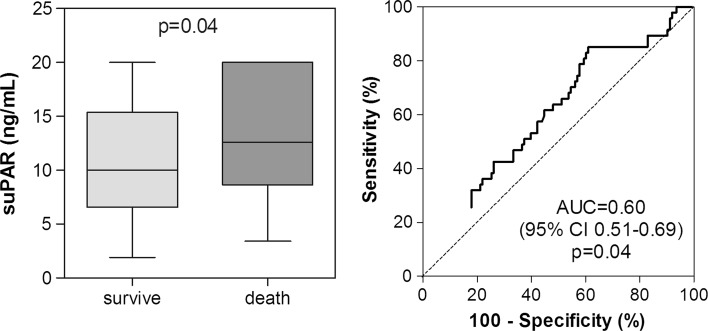
Prognostic power of suPAR in patients with sepsis. Box plot graphics and ROC curve analyses showing the prognostic power of suPAR for mortality on admission in patients with sepsis. *AUC* area under the curve. The *p* value indicates the level of statistical significance. Adapted with permission from [25]

Systemic levels of suPAR were significantly higher in critically ill patients with fatal outcomes compared to patients who survive critical illness [20, 21, 23, 25–27]. Although systemic levels of suPAR were consistently higher in non-survivors on admission as well as on day 3 and 7, the AUC for suPAR to predict ICU mortality in general ICU patients remained moderate (Fig. 3) [25]. Similar findings were seen in patients with sepsis (Fig. 4) [25]. In patients who fulfilled at least two criteria for SIRS, an AUC of 0.80 (cutoff value 6.61 μg/l, sensitivity 89 %, specificity 63 %) for short-term mortality and an AUC of 0.69 (cutoff value 6.61 μg/l, sensitivity 89 %, specificity 63 %) for long-term mortality was found [23]. Of note, 64 % of these patients suffered from bacterial infection, and 15 % had positive blood cultures. In addition, the AUC in patients with positive blood cultures was 0.75 (cutoff value 9.25 ng/ml, sensitivity 79 %, specificity 68 %) for 1-month mortality and AUCs of 0.80 (cutoff value 8.3 ng/ml, sensitivity 71 %, specificity 78 %) and 0.84 (cutoff value 11 ng/ml, sensitivity 83 %, specificity 76 %) for hospital mortality [20, 21, 27]. Notably, changes in systemic levels of suPAR over the first few days in the ICU did not appear to differ between survivors and non-survivors [25].

As a single biological marker, suPAR was superior in predicting mortality compared to other frequently used biological markers, including CRP, PCT and sTREM-1 [20, 21, 23, 25] (Table 3). In addition, the prognostic performance of suPAR improved when combined with other biological markers or age [23] (Table 3). While the ability of suPAR to predict mortality was poor compared to the conventional simplified acute physiology score (SAPS) II [23, 25], the prognostic performance of SAPS II improved when combined with suPAR levels [23] (Table 3).

**Table 3:** Prognostic value of suPAR to predict mortality as compared to other biological markers and disease severity scores

Ability to predict	Parameter	AUC (95 % CI)	Cutoff	Sensitivity ( %)	Specificity ( %)	References
General intensive care unit population						
ICU mortality	suPAR	0.67 (0.54–0.80)	–	–	–	[25]
	CRP	0.54 (0.40–0.68)	–	–	–	
	PCT	0.58 (0.46–0.71)	–	–	–	
	SAPS II	0.83 (0.74–0.91)	–	–	–	
	APACHE II	0.60 (0.45–0.74)	–	–	–	
Long-term mortality^a^	suPAR	0.67 (0.55–0.78)	–	–	–	[25]
	CRP	0.55 (0.43–0.67)	–	–	–	
	PCT	0.62 (0.50–0.74)	–	–	–	
	SAPS II	0.73 (0.62–0.84)	–	–	–	
	APACHE II	0.63 (0.51–0.75)	–	–	–	
Patients with infectious diseases^b^						
30-Day mortality	suPAR	0.80 (0.69–0.92)	6.61 μg/L	89	63	[23]
	sTREM-1	0.69 (0.52–0.86)	9.00 μg/L	100	36	
	MIF	0.65 (0.46–0.84)	1.27 μg/L	78	54	
	suPAR + age	0.92 (0.86–0.97)	3.43	100	78	
	suPAR + sTREM–1 + MIF	0.84 (0.70–0.98)	2.40	67	93	
	suPAR + sTREM–1 + MIF + age	0.93 (0.88–0.98)	3.40	100	81	
	SAPS II	0.89 (0.80–0.98)	22.5	100	68	
	SOFA	0.80 (0.65–0.94)	4.5	44	95	
	suPAR + SAPS II	0.93 (0.86–1.00)	–	–	–	
180-Day mortality	suPAR	0.69 (0.57–0.81)	6.61 μg/L	68	64	[23]
	sTREM-1	0.69 (0.58–0.80)	9.00 μg/L	95	38	
	MIF	0.54 (0.39–0.68)	0.81 μg/L	42	72	
	suPAR + age	0.86 (0.79–0.94)	4.62	79	83	
	suPAR + sTREM–1 + MIF	0.72 (0.59–0.84)	1.87	58	83	
	suPAR + sTREM–1 + MIF + age	0.87 (0.79–0.94)	4.55	79	84	
	SAPS II	0.91 (0.86–0.96)	22.5	100	73	
	SOFA	0.75 (0.64–0.86)	1.5	74	61	
	suPAR + SAPS II	0.92 (0.87–0.97)	–	–	–	
Bacteremic patients						
1-Month mortality	suPAR	0.75 (0.62–0.89)	9.25 ng/ml	79	68	[20]
	CRP	0.60 (0.44–0.75)	–	–	–	
Hospital mortality	suPAR	0.80 (0.62–0.90)	8.3 ng/mL	71	78	[21]
	CRP	0.76 (0.59–0.86)	1360 nmol/L	82	69	

*APACHE II* Acute Physiology and Chronic Health Evaluation II score, *CRP* C-reactive protein, *MIF* macrophage migration inhibitory factor, *PCT* procalcitonin, *SAPS II* Simplified Acute Physiology Score II, *SOFA* Sequential Organ Failure Assessment, *sTREM*-*1* soluble triggering receptor expressed on myeloid cells type 1

^a^Long-term follow-up period: median 348 days (range 29–884)

^b^Of these patients, 64 % suffered from bacterial infection, and 15 % had positive blood cultures

Levels of suPAR did not seem predictive of length of ICU stay [25]. High systemic levels of suPAR were associated with the need for ICU admission, and need for vasopressors and mechanical ventilation [27].

### Correlation between suPAR and organ failure markers and disease severity scores

In non-septic and septic patients, systemic levels of suPAR correlated with several markers of organ dysfunction, including creatinine, urea and cystatin C (kidney), bilirubin, gamma glutamyl transpeptidase, alkaline phosphatase and albumin (liver), and INR and antithrombin (coagulation) [25]. Similar associations were described for blood culture-positive bacteremic patients [27].

In the general ICU population, systemic levels of suPAR correlated with Acute Physiology and Chronic Health Evaluation (APACHE) II scores, SAPS II, and Sequential Organ Failure Assessment (SOFA) scores [25]. In patients with sepsis, systemic levels of suPAR also correlated with APACHE II scores and SAPS II, but not with SOFA scores [25]. Of note, in patients with urosepsis no correlation with APACHE II scores was found [28]. In bacteremic patients, however, high suPAR levels were associated with high SOFA scores [27].

### Systemic levels of suPAR after initiation of antimicrobial treatment

Three studies evaluated levels of suPAR after the initiation of treatment [25, 27, 28]. Systemic levels of suPAR remained elevated for hours in patients with urosepsis [28] and for at least the first week in a general ICU population [25]. In bacteremic patients, systemic levels of suPAR tended to decline only after several weeks [27].

### Factors that influence levels of suPAR and/or its performance

Some studies showed that the type of assay used to measure suPAR as well as age of patients and underlying diseases affected levels of suPAR and/or its performance.

The types of test used for measurement of suPAR are decribed in Table 2. In one study the suPARnostic™ (Virogates, Copenhagen, Denmark) assay was compared with the Luminex assay in the same cohort of patients and showed a better prognostic performance (AUC of 0.80 vs. 0.68 in predicting short-term mortality; AUC of 0.69 vs. 0.54 in predicting long-term mortality) [23]. Another study found a correlation coefficient between suPAR concentrations obtained with a Luminex (8-plex) assay and a suPAR ELISA of 0.95 with a 95 % limits of agreement between 99–140 % [24].

Studies that investigated systemic levels of suPAR in relation to age are contradictory [20, 21, 25–27]. Two studies found no correlation between systemic levels of suPAR and age [25, 26]. However, two other studies suggested higher levels of suPAR in elderly patients [20, 27], and one study showed lower levels in very old patients [21]. Of note, in patients with bacteremia with age ≥75 years, differences in systemic levels of suPAR between surviving and non-surviving patients were not significant [21].

Underlying diseases may affect levels of suPAR. In patients with bacteremia levels of suPAR were higher in patients with chronic alcohol abuse or liver disease, while levels were lower in patients with cancer [26, 27]. Interestingly, after adjusting for possible confounders, including age and underlying diseases, systemic levels of suPAR remained independently prognostic for mortality [20, 25–27].

## Discussion

This systematic review shows that although systemic levels of suPAR are elevated with SIRS, bacteremia, and sepsis, its diagnostic value is low, as suPAR is a non-specific marker of inflammation. Systemic levels of suPAR, however, do have prognostic value, with higher levels being associated with increased mortality. Systemic levels of suPAR correlate positively with severity-of-disease classification scores. Systemic levels of suPAR also correlate positively with several markers of organ dysfunction. After initiation of therapy, levels of suPAR decline only on long-term follow-up.

Ideally, biological markers of sepsis should differentiate among bacterial, viral and fungal infection, and between systemic sepsis and local infection. Over 150 biological markers have been clinically evaluated for use in sepsis [30]. Relatively few biological markers, however, have been used for diagnosing (the type of) sepsis, and none has sufficient sensitivity or specificity to be used routinely in daily practice [30]. C-reactive protein and procalcitonin are the most widely used values, despite their limited ability to distinguish sepsis from other inflammatory conditions [30]. Our review showed suPAR to have low diagnostic value in patients with SIRS, bacteremia, or sepsis, even lower than CRP, PCT, and sTREM-1.

The majority of the biological markers investigated in septic patients have been assessed according to their prognostic value [30]. Traditional markers such as fever, white blood cell count, and CRP levels are not reliable for assessing disease severity and mortality risk [30, 31]. Procalcitonin seems to be an improvement on these markers, but is not ideal [32–34]. Although PCT has repeatedly been shown to have prognostic value in critically ill patients, the value of a single level on admission is limited [35]. The compiled data in this paper suggest that suPAR has superior prognostic value compared to commonly used biological markers, including PCT. Moreover, in contrast to most other markers, circadian changes in plasma levels of suPAR are minimal [28, 36]. Measurement is therefore largely independent of the sampling schedule, improving the potential of suPAR as a marker in clinical routine.

It has been suggested that suPAR is involved in the plasminogen-activating pathway, inflammation, and the modulation of cell adhesion, migration, and proliferation [37]. Soluble urokinase-type plasminogen activator receptor derives from proteolytic cleavage and release from cell membrane-bound urokinase plasminogen activator receptor (uPAR). Both membrane-bound and soluble uPARs have been shown to bind to integrins [38, 39], and have been proposed to be involved in cell adhesion and proliferation. The soluble form of uPAR has been reported to have direct chemotactic properties, which may facilitate recruitment of inflammatory cells such as neutrophils and monocytes [40, 41], and the mobilization of hematopoietic stem cells [42]. In addition to its role in adhesion and migration, suPAR has recently been shown to inhibit neutrophil efferocytosis [43], while the membrane-bound form of uPAR has been shown to facilitate phagocytosis of bacteria [44]. Impaired engulfment of apoptotic neutrophils or bacteria has been associated with poor outcome in preclinical models of sepsis [44, 45]. Cleavage of uPAR may therefore reflect a functional impairment of the host defense rather than a surrogate marker of inflammation, which might explain the higher prognostic value of suPAR compared to other biological markers.

Although suPAR alone did not perform as well as the SAPS II score, this does not necessarily preclude its use in prognostication. APACHE II score, SAPS II, SOFA score, and other scoring systems estimating the risk of mortality have become increasingly popular in the field of research with critically ill patients over the last decades. However, in clinical practice these scoring systems have important limitations. Data collection requires multiple laboratory measurements and the computation of multiple variables, and is labor intensive and expensive [46–48]. Therefore, the application of these scoring systems may be limited, particularly when health care is subject to financial constraint. Soluble urokinase-type plasminogen activator receptor may have other important advantages. Only one blood sample instead of multiple clinical and laboratory measurements are needed. Measurement of suPAR can be performed using a simple ELISA. In addition, suPAR is stable in plasma samples subjected to repeated freeze-thaw procedures [49], increasing its practicality as a practical biological marker. Thus, based on the findings that systemic levels of suPAR are a strong and robust marker of mortality risk, one could speculate that suPAR will eventually serves as a quick, technically simple and inexpensive alternative to the current sophisticated severity-of-disease classification systems. Future studies are needed to address this hypothesis.

The usefulness of suPAR in mortality prediction of individual patients is uncertain. One can hypothesize that specific therapeutic strategies should be restricted to patients with a certain level of suPAR as an alternative to APACHE II or SAPS II scores. Risk stratification and prediction of outcome can be used for safe decision making on the need for hospitalization or ICU admission and identifying patients at low risk suitable for outpatient management. Thus, suPAR may eventually help to triage patients. Also, predicted mortalities can be averaged for groups of patients in order to specify the group’s morbidity. However, conclusions should be drawn with some caution. First, patient numbers in studies of suPAR are still very low. More and larger studies are needed to better define the prognostic power of suPAR in critically ill patients.

Notably, systemic levels of suPAR remain elevated long after clinical recovery and only decline after several weeks. Therefore, the use of suPAR as a biological marker for guiding therapy is probably limited. However studies addressing this issue are lacking.

Importantly, the type of assay used to measure suPAR, as well as age and presence or absence of underlying diseases all influence suPAR levels. The difference in prognostic performance between different assays can be explained by the fact that the Luminex assay uses a polyclonal detection antibody, whereas the suPARnostic™ assay uses monoclonal antibodies selected because of their superior clinical value in HIV disease progression [23]. On the other hand, the Luminex assay has its advantage in measuring multiple analytes at the same time [24]. With the prognostic value of suPAR increasing in combination with other markers, this might compensate for the slightly impaired performance.

The finding that age as well as underlying disease influences the systemic level of suPAR is of limited relevance as both age and underlying diseases are known to increase mortality risk in critically ill patients [50, 51]. Systemic levels of suPAR remained independently prognostic for mortality after adjusting for age and/or underlying diseases [20, 25–27]. As with other markers such as CRP and PCT, experience will eventually dictate the value of suPAR levels in diverse clinical situations.

Of interest, suPAR is not only present in human plasma or serum, but can also be found in other body fluids, including urine, cerebrospinal fluid [37], and pleural, pericardial, and peritoneal fluids [17]. The number of studies investigating the value of suPAR in body fluids other than plasma or serum, however, is very limited. It would be interesting to evaluate the value of local levels of suPAR in other body fluids, i.e., in bronchoalveolar lavage fluid of patients with frequent pulmonary complications, such as acute lung injury or ventilator-associated pneumonia.

Finally, this review has limitations. An important limitation is that not all studies used the ACCP/SCCM criteria for the diagnosis of bacteremia, SIRS, and sepsis. Differences in used definitions may hamper interpretation of the data. However, since the aim of this review is to describe the value of suPAR in patients with systemic infection or inflammation and not to compare patients with SIRS with patients with bacteremia or sepsis, overlap between these groups may not hamper interpretation of the results.

The most common limitation of any systematic review is publication bias. Unpublished materials were not found and thus not used. Another limitation is the small number of studies that could be included. Soluble urokinase-type plasminogen activator receptor is a relatively new marker, and the number of publications in critically ill patients is still low. Also, studies on the prognostic value of suPAR were very restricted as they focused only on mortality. No conclusions can be drawn on the prognostic value of suPAR on other clinical outcomes, such as length of ICU and hospital stay, and duration of mechanical ventilation.

## Conclusions

Soluble urokinase-type plasminogen activator receptor seems a promising prognostic marker in critically ill patients. Currently, studies are limited to the predictive potential to estimate the mortality risk in observational designs. Future studies should demonstrate whether prognostic assessment translates into better clinical outcomes and a higher quality of patients care.
